# Reliability model of the security subsystem countering to the impact of typed cyber-physical attacks

**DOI:** 10.1038/s41598-022-17254-4

**Published:** 2022-07-27

**Authors:** Viacheslav Kovtun, Ivan Izonin, Michal Gregus

**Affiliations:** 1 Vinnytsia National Technical University, Vinnytsia, 21000 Ukraine; 2 Lviv Polytechnic National University, Lviv, 79013 Ukraine; 3 Comenius University in Bratislava, Bratislava, 820 05 Slovak Republic

**Keywords:** Computer science, Information technology

## Abstract

The article's main contribution is the description of the process of the security subsystem countering the impact of typed cyber-physical attacks as a model of end states in continuous time. The input parameters of the model are the flow intensities of typed cyber-physical attacks, the flow intensities of possible cyber-immune reactions, and the set of probabilities of neutralization of cyber-physical attacks. The set of admissible states of the info-communication system is described taking into account possible variants of the development of the modeled process. The initial parameters of the model are the probabilities of the studied system in the appropriate states at a particular moment. The dynamics of the info-communication system's life cycle are embodied in the form of a matrix of transient probabilities. The mentioned matrix connects the initial parameters in the form of a system of Chapman's equations. The article presents a computationally efficient concept based on Gershgorin's theorems to solve such a system of equations with given initiating values. Based on the presented scientific results, the article proposes the concept of calculating the time to failure as an indicator of the reliability of the info-communication system operating under the probable impact of typical cyber-physical attacks. The adequacy of the model and concepts presented in the article is proved by comparing a statically representative amount of empirical and simulated data. We emphasize that the main contribution of the research is the description of the process of the security subsystem countering the impact of typed cyber-physical attacks as a model of end states in continuous time. Based on the created model, the concept of computationally efficient solution of Chapman's equation system based on Gershgorin's theorems and calculating time to failure as an indicator of the reliability of the info-communication system operating under the probable impact of typed cyber-physical attacks are formalized. These models and concepts are the highlights of the research.

## Introduction

A Cyber-Physical Attack (CPA) is an intentional or unintentional impact on the computing or communication infrastructure of the target system that causes a failure in the control of sensors or actuators. A CPA often exploits vulnerabilities in computing or communication components of relevant systems. For example, suppose an attacker manages to gain control over automated elements of municipal infrastructure, medical implants, and self-driving vehicles. In that case, he can cause damage to the physical dimension from the information dimension, endangering both material values and human lives. Today's society is so dependent on computer and network systems that CPAs are now considered a key threat to critical national infrastructures and a real threat to ordinary citizens^1–6^.

The success of a CPA guarantees previous research aimed at identifying active vulnerabilities and relevant entry points into the target process. To assess the scale of the problem, we list only potential entry points^7–9^:

- radio communication between Remote Terminal Units (RTUs) / programmable logic controllers (PLCs) and sensors/actuators / Supervisory Control And Data Acquisition (SCADA) servers;
- control network formed by SCADA servers and operator workstations;
- communication gateway/channel between the control system and the corporate network (for example, the switching point between the primary and secondary archivers);
- corporate networks;
- Internet and corporate networks of partners;
- Wi-Fi network and related network equipment
- mobile and desktop service applications for the control of the appropriate process.

Paradoxically, although Cyber-Physical Systems' (CPS) system and application software is not intended for general use, there are no specialized "cyber-physical" security mechanisms. Universal tools are used^10–13^: authentication, access control, firewall, antivirus software, application/thread safelists, cryptography, and integrity control. In addition, this is even though the priority for CPSs is integrity and availability, rather than confidentiality (as for general-purpose computer systems). The latter fact necessitates a radically specific approach to forming security policy for CPSs. However, while teams of cyber-security experts are addressing this issue, the ***urgent task*** is to assess the reliability of existing CPSs, which is ensured by the ability of existing protection mechanisms to counter current types of CPAs.

Reliability theory^14–19^ is a powerful, constantly evolving branch of theoretical science. The basic mathematical apparatus underlying it includes the provisions of probability theory and mathematical statistics, random process theory, queuing theory, mathematical logic, graph theory, optimization theory, and so on.

These methodologies form a toolkit for analyzing the performance of the studied information systems for a finite censored period. The analysis takes place in the context of determining:

1. The time between failures in the studied system;
2. The number of failures in the studied system for the censored period of its operation;
3. The reaction of the studied system to the provoked failures;
4. The response of the studied system to complex test effects.

Models^6–9^ are focused on the description of the first performance indicator. They are based on the mathematical apparatus of time series analysis. Their purpose is to identify the parameters of the statistical distribution, which best describes the period between failures in the operation of the studied system. The adequacy of such models is determined by the representativeness of the data sample that characterizes the studied system's operation. When formalizing such models, only the fact of failure is taken into account without analyzing the causes of its occurrence and possible consequences.

Models^10–13^ are focused on the description of the second performance indicator. It is assumed that a particular distribution law (most often Poisson's) with a continuous or discrete intensity function describes the stochastic parameter, which characterizes the number of time failures. The latter is determined by the results of static analysis of operational data. The disadvantages of this type of model are similar to those mentioned above.

Models^14–17^ are focused on the description of the third performance indicator. The data for analysis in these models are:

- the number of failures in the studied system for the censored period, which was caused by unknown negative impacts;
- the number of failures in the operation of the studied system during the censored period, which was caused by negative impacts, and the mechanisms of counter-action which were embedded in the studied system at the stage of its design.

Data analysis is carried out by combinatorics and maximum likelihood methods. Such models are more informative but are still based on information, some of which were collected because of uncontrolled experiments.

Models^18–22^ are focused on the description of the fourth performance indicator based solely on the results of controlled experiments. Considering that the causes of failures are usually interrelated, models of this type are based on the mathematical apparatus of Markov chains. It allows considered the multithreading in the operation of the studied system and the heterogeneity of the process of its recovery after a failure. Semi-Markov models more accurately describe the behavior of real information systems because the process of recovery of the first ones after failures can be characterized not only by the exponential distribution functions. The structural features of the studied system in this modeling approach can be considered in the graph of the flow of control, which brings the model closer to the described process. This qualitatively distinguishes the Markov approach from, for example, a nonparametric neural network^23,24^, in which the structural features of the studied system are ignored.

A notable element of the methodological apparatus of the reliability theory, particularly info-communication systems, is the structural-logical method^20–23^. The method describes the studied system as a topology of interacting elements (devices, software services, operators), the set of which uniquely identifies the original studied object. Analytically, the relationships in the topology are characterized by the corresponding functions of the algebra of logic. The same functions in the transition to probabilistic or deterministic structural-logical models become the basis for formalizing the criteria for identifying the set of states of the studied system. Quantitative indicators of reliability based on the structural-logical model of the studied system are determined by replacing the minimum disjunctive normal form of logical functions with probabilistic or deterministic characteristics with a simultaneous transition from logical to arithmetic operations on them.

Graphical interpretation of structural-logical models is the corresponding schemes of functional integrity^22–25^, depicted in the form of block diagrams, trees of faults or (and) events, the "bow-tie" techniques, and so on. An algebra of groups of incompatible events in the paradigm of the general logical-probabilistic method^20,23,24,26^ has been developed to remove the binary constraint (the stay of the model element in only one of two defined states), characteristic of structural-logical models. Note also that the fault tree analysis method is the basis for forming dynamic fault trees (have a wide range of logical operators) or generalized fault trees (possible further conversion to Bayesian networks, Petri nets, etc.) However, these add-ons are not used in practice due to their excessive complexity in implementing and interpreting the initial results. But, in the authors' opinion, the analysis of generalized fault trees seems promising due to the possibility of their direct integration with certain methods of machine learning.

However, the authors consider that the mathematical apparatus of Markov chains^23,24,27–31^ is optimal for analyzing the reliability of CPSs taking into account their infrastructural and operational features^1–9^. Close analogs are the reliability models of information systems described in articles^24,27,29–31^, formalized based on discrete Markov chains. These models represent the info-communication system as a system with failures and recoveries. The strength of the mentioned research is the mathematically correct stochastic characteristic of the states of the studied system and the analytically determined functional connection of the assessment of the reliability of the studied system with the relaxation time of the Markov chain. However, the apparent theoretical orientation of the scientific results presented in these articles is limited to their application. Also, the finite parametric space used in these investigations characterizes the studied processes in terms of probability theory and mathematical statistics rather than the theory of reliability. Finally, describing the studied process in discrete time presented in the mentioned articles introduces inaccuracy in assessing the reliability indicators.

Given the strengths and weaknesses of these analogs, we formulate the necessary attributes of our scientific research.

The ***research object*** is the process of the security subsystem of the info-communication system countering the impact of typed CPAs.

The ***research subject*** is the provisions of the theory of Markov processes, the theory of differential equations, the theory of probability and mathematical statistics, and the theory of experimental planning.

The ***aim*** of the research is the analytical formalization of estimating the reliability of the info-communication system on the model of the security subsystem, countering the impact of typed CPAs.

The ***objectives*** of the research are

1. The analytical formalization of the process of the security subsystem countering the impact of typed CPAs as a Markov chain in continuous time;
2. The analytical formalization of the computationally efficient concept of calculating the probabilities of realization of states in which the studied info-communication system can be at any moment;
3. The analytical formalization of the concept of calculating the time to failure as an indicator of the reliability of the info-communication system, operating under conditions of the probable impact of typified CPAs;
4. Proving the adequacy and demonstration of the functionality of the created mathematical apparatus.

The ***main contribution*** of the research is the description of the process of the security subsystem countering the impact of typed CPAs as a model of end states in continuous time. Based on the created model, the concept of computationally efficient solution of Chapman's equation system based on Gershgorin's theorems and calculating time to failure as an indicator of the reliability of the info-communication system operating under the probable impact of typed CPAs are formalized. These models and concepts are the ***highlights*** of the research.

## Models and methods

### Research statement and the proposed model

Consider the process of functioning of the info-communication system, which is potentially vulnerable to *n* CPAs. In general, the ability to counter CPAs is determined by the reliability of the target system, which is provided by the architecture and settings of its security subsystem. We formalize a mathematical model that allows us to quantify the process of the security subsystem confrontation to the impact of the typed CPAs.

Let the sequence of occurrence of an arbitrary *i*-th CPA be described by a Poisson flow of events with intensity $\lambda_{i}$. The oncoming Poisson flow of the cyber-immune reaction is characterized by the intensity $\mu_{i}$ and the probability of neutralization of the *i*-th CPA *r*_*i*_. When $i \in \left\{ {\overline{1,n} } \right\}$ the input parameters of the model are ordered in the form of:

- the set of intensities of CPAs $\Lambda = \left\{ {\overline{{\lambda_{1} ,\lambda_{n} }} } \right\}$, $\lambda_{i} \ge 0$;
- the set of intensities of cyber-immune reactions ${\rm M} = \left\{ {\overline{{\mu_{1} ,\mu_{n} }} } \right\}$, $\mu_{i} \ge 0$;
- the set of probabilities of neutralizing the corresponding CPAs $R = \left\{ {\overline{{r_{1} ,r_{n} }} } \right\}$, $0 \le r_{i} \le 1$.

The functioning of the CPS in potentially aggressive conditions will be presented in terms of the finite state model. Let us denote by *s*_0_ the serviceable state in which the system is, on which no CPA is carried out. The antagonist of the serviceable state will be the state of failure $s_{n + 1}$ in which the system is, on which the CPA was not successful. Intermediate between these polar states will be the states of confrontation *s*_*i*_. The system is in a state of confrontation *s*_*i*_ when it is under the CPA of *i*-th type. The duration of the state of confrontation *s*_*i*_ is a stochastic quantity with a Poisson distribution with parameter $\mu_{i}$. Depending on the result of the cyber-immune reaction, the system from the state of confrontation *s*_*i*_ either with the probability *r*_*i*_ transits to the serviceable state *s*_0_ or with the probability $\left( {1 - r_{i} } \right)$ transits to the state of failure $s_{n + 1}$. Assume that the model characterizes the functioning of the security subsystem of the CPS of critical use, the transition of which into a state of failure marks the end of the life cycle (return to the serviceable state is impossible).

**Figure 1:**
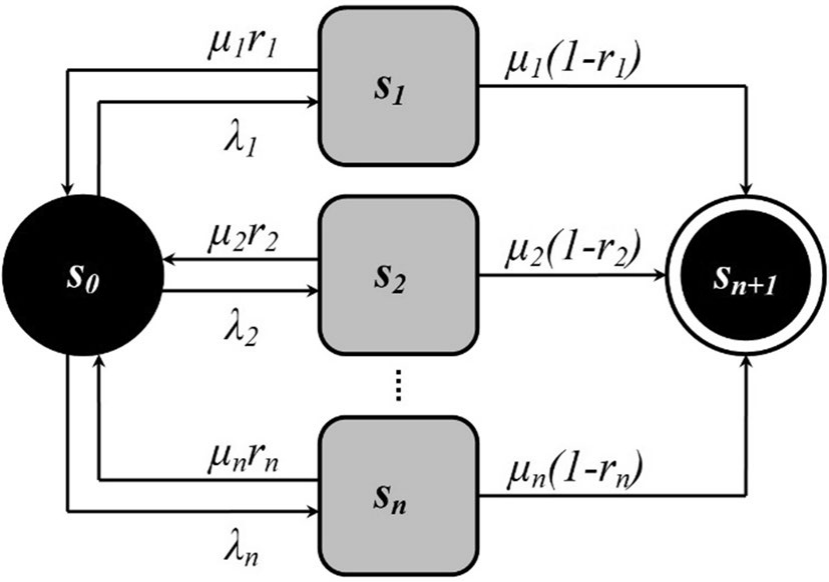
UML-state diagram of the model of the studied process.

The described model of finite states is represented in graphical form by a UML state diagram (see Fig. 1).

The probability that the studied cyber*physical system at a time *t* is in the state *s*_*i*_ is denoted as $p_{i} \left( t \right)$, $i \in \left\{ {\overline{0,n + 1} } \right\}$. These probabilities can be determined in general by solving a system of ordinary differential equations known as Chapman's equations^32,33^. We specify the analytical form of these equations for the model of the studied process, presented graphically in Fig. 1. We obtain:

1 $$\begin{aligned} \frac{{dp_{0} \left( t \right)}}{dt} & = - \lambda_{0} p_{0} \left( t \right) + \sum\limits_{j = 1}^{n} {\mu_{j} r_{j} p_{j} \left( t \right)} , \\ \frac{{dp_{i} \left( t \right)}}{dt} & = \lambda_{i} p_{0} \left( t \right) - \mu_{i} p_{i} \left( t \right),\;\;i = \overline{1,n} , \\ \frac{{dp_{n + 1} \left( t \right)}}{dt} & = \sum\limits_{j = 1}^{n} {\mu_{j} \left( {1 - r_{j} } \right)p_{j} \left( t \right)} , \\ \end{aligned}$$

where $$\lambda_{0} = \sum\nolimits_{i = 1}^{n} {\lambda_{i} }$$.

The probabilities $p_{i} \left( t \right)$ can be unambiguously determined based on the system of Eq. (1) only by setting their initiating values. Let

2 $$p_{0} \left( 0 \right) = 1,\quad p_{j} \left( 0 \right) = 0,\quad j = \overline{1,n + 1} ,$$

i.e. at the time, $t = 0$ the CPS is in a serviceable state.

For compactness, we present a system of Eq. (1) in matrix form:

3 $$\frac{{d{\text{p}}\left( t \right)}}{dt} = \Pi \cdot {\text{p}}\left( t \right),$$

where ${\text{p}}\left( t \right) = \left\{ {p_{0} \left( t \right),p_{1} \left( t \right), \ldots ,p_{n + 1} \left( t \right)} \right\}$ is the set of probabilities of realization of the corresponding state for the studied system, and $\Pi$ is a square matrix of probabilities of transitions of dimension $\left( {n + 2} \right) \times \left( {n + 2} \right)$, the sum of the elements of each row of which is zero:

4 $$\Pi = \left( {\begin{array}{*{20}c} { - \lambda_{0} } & {\lambda_{1} } & {\lambda_{2} } & \ldots & {\lambda_{m} } & 0 \\ {\mu_{1} r_{1} } & { - \mu_{1} } & 0 & \ldots & 0 & {\mu_{1} \left( {1 - r_{1} } \right)} \\ {\mu_{2} r_{2} } & 0 & { - \mu_{2} } & \ldots & 0 & {\mu_{2} \left( {1 - r_{2} } \right)} \\ \ldots & \ldots & \ldots & \ldots & \ldots & \ldots \\ {\mu_{n} r_{n} } & 0 & 0 & \ldots & { - \mu_{n} } & {\mu_{n} \left( {1 - r_{n} } \right)} \\ 0 & 0 & 0 & \ldots & 0 & 0 \\ \end{array} } \right).$$

Thus, we presented the process of the security subsystem confronting the typed CPAs in the form of a Markov chain in continuous time, which connects the set of input data $\left\langle {\Lambda ,{\rm M},R} \right\rangle$ with the system of Chapman's Eq. (1) using a matrix (4) taking into account the initiating values (2). The initial parameters of the model are the set of probabilities of realization of the corresponding states for the studied system in the form (3).

### The concept of solving the Chapman equation system for the model of the studied process

In general, the solution of the system of Eq. (1), taking into account the initiating values (2), is relatively non-trivial. However, the description based on the proposed model of individual situations, typical for the life cycle of the CPS, may be the preamble, the existence of which will facilitate the further perception of readers of the material in this section.

Based on the model (1), we describe the CPS's situation in a serviceable state *s*_0_. In this situation: $\lambda_{i} = 0\forall i \in \left\{ {\overline{1,n} } \right\}$. Under such conditions, the solution of the Cauchy problem for the system of Eq. (1) can be represented as $p_{0} \left( t \right) = 1$, $p_{j} \left( t \right) = 0$, $j = \overline{1,n + 1}$. The resulting solution illustrates the obvious fact—if no CPA is carried out on the system, the last one is in the serviceable state.

An antagonist is when the security subsystem lacks a specialized protective mechanism to counter a typified CPA. Let's assume that $r_{i} = 0\forall i$. If no parameter $\mu_{i}$ coincides with $\lambda_{0}$, then we can analytically define the solutions of the corresponding first-order Kolmogorov Eq. (1), which describe the state graph shown in Fig. 1, in the form of such expressions:

$$\begin{gathered} p_{0} \left( t \right) = \exp \left( { - \lambda_{0} t} \right), \hfill \\ p_{i} \left( t \right) = \frac{{\lambda_{i} }}{{\lambda_{0} - \mu_{i} }}\left( {\exp \left( { - \mu_{i} t} \right) - \exp \left( { - \lambda_{0} t} \right)} \right),\;\;i = \overline{1,n} , \hfill \\ p_{n + 1} \left( t \right) = 1 - \exp \left( { - \lambda_{0} t} \right) - \sum\limits_{i = 1}^{n} {\frac{{\lambda_{i} }}{{\lambda_{0} - \mu_{i} }}} \left( {\exp \left( { - \mu_{i} t} \right) - \exp \left( { - \lambda_{0} t} \right)} \right). \hfill \\ \end{gathered}$$

The resulting expressions are formulated for the initial conditions defined in the form (2). From the obtained solution, it is seen that if the protective mechanism such as CPA does not match, the probability of the studied system in the serviceable state decreases exponentially, and the probability of its transition to the failure state $s_{n + 1}$, on the contrary, increases towards one.

We now turn to solve the system of Eq. (1). Laplace transforms are usually used to solve the system of Chapman's equations with constant coefficients. We managed to propose a computationally efficient method based on the use of eigenvectors and eigenvalues of the transition probability matrix (4). Let's substantiate this thesis.

Prove that all real numbers of the matrix (4) belong to the interval $\left[ { - 2\gamma ,0} \right]$, where $\gamma = \max \left\{ {\mu_{1} ,\mu_{2} , \ldots ,\mu_{n} ,\lambda_{0} } \right\}$. The results presented in^34^ confirm that the eigenvalues of the matrix of the form (4) belong to the set of real numbers. Our statement about the interval $\left[ { - 2\gamma ,0} \right]$ is based on Gershgorin's theorems^34,35^. Indeed, on their basis, it can be stated that all eigenvalues of the matrix (4) belong to the interval formed by combining the corresponding segments:

5 $$\left[ { - 2\mu_{1} ,0} \right] \cup \left[ { - 2\mu_{2} ,0} \right] \cup \ldots \cup \left[ { - 2\mu_{n} ,0} \right] \cup \left[ { - 2\lambda_{0} ,0} \right].$$

From expression (5) it follows that each eigenvalue of the matrix (4) will belong to the segment $\left[ { - 2\gamma ,0} \right]$, where $\gamma = \max \left\{ {\mu_{1} ,\mu_{2} , \ldots ,\mu_{n} ,\lambda_{0} } \right\}$. Moreover, the matrix (4) always has a zero eigenvalue $\sigma_{0} = 0$, because it has a zero string.

Based on expression (5), we define the spectrum of the matrix (4) as

6 $$spec\left( \Pi \right) = \left\{ {\sigma_{0} = 0, - \left| {\sigma_{1} } \right|, - \left| {\sigma_{2} } \right|, \ldots , - \left| {\sigma_{n + 1} } \right|} \right\},$$

where $\sigma_{1} ,\sigma_{2} , \ldots ,\sigma_{n + 1}$ are negative real numbers. Next, we consider the spectrum (6) simple.

From the general theory of differential equations adapted to Chapman's system of equations, it is known that system (3) has *n* + 2 linear independent solutions of the form

$${\text{p}}_{0} \left( t \right) = {\text{c}}_{0} ,\quad {\text{p}}_{1} \left( t \right) = {\text{c}}_{1} \exp \left( {\sigma_{1} t} \right),\quad {\text{p}}_{2} \left( t \right) = {\text{c}}_{2} \exp \left( {\sigma_{2} t} \right), \ldots ,{\text{p}}_{n + 1} \left( t \right) = {\text{c}}_{n + 1} \exp \left( {\sigma_{n + 1} t} \right),$$

where ${\text{c}}_{l}$ is the left eigenvector of the matrix (4), which corresponds to the eigenvalue $\sigma_{l}$, $l = \overline{0,n + 1}$.

Let's choose eigenvectors ${\text{c}}_{l}$ so that the condition

7 $$\sum\limits_{l = 0}^{n + 1} {{\text{c}}_{l} } = {\vec{\text{e}}}$$

is satisfied, where ${\vec{\text{e}}}$ is the vector with *n* + 2 elements of the form $\left( {1,0, \ldots ,0} \right)$. Then the solution of the system of Chapman's equations of the form (1) with the initiating values (2) can be analytically expressed as

8 $$\begin{aligned} {\text{p}}\left( t \right) & = \sum\limits_{l = 0}^{n + 1} {{\text{c}}_{l} \exp \left( {\sigma_{l} t} \right)} = {\text{c}}_{0} + {\text{c}}_{1} \exp \left( {\sigma_{1} t} \right) \\ & \quad + {\text{c}}_{2} \exp \left( {\sigma_{2} t} \right) + \cdots + {\text{c}}_{n + 1} \exp \left( {\sigma_{n + 1} t} \right). \\ \end{aligned}$$

Rewrite expression (8) as follows:

9 $$p_{i} \left( t \right) = \delta_{i,n + 1} + \sum\limits_{l = 1}^{n + 1} {c_{l,i} \exp \left( {\sigma_{l} t} \right)} ,\quad i = \overline{0,n + 1} ,$$

where $\delta$ is a Kronecker symbol, $c_{l,i}$ is the *i*-th element of the eigenvector ${\text{c}}_{l}$. Also, when formalizing expression (9), we took into account the fact that the eigenvector c_0_ of length $n + 2$, which corresponds to the eigenvalue $\sigma_{0} = 0$, is defined as $\left( {0, \ldots ,0,1} \right)$.

Initial values (2) can also be represented in terms of eigenvectors. Assuming ${\text{c}}_{l} = \left( {c_{l,i} } \right)$, we write:

10 $$\sum\limits_{l = 1}^{n + 1} {c_{l,i} } = \delta_{0,i} - \delta_{n + 1,i} ,\quad i = \overline{0,n + 1} .$$

**Figure 2:**
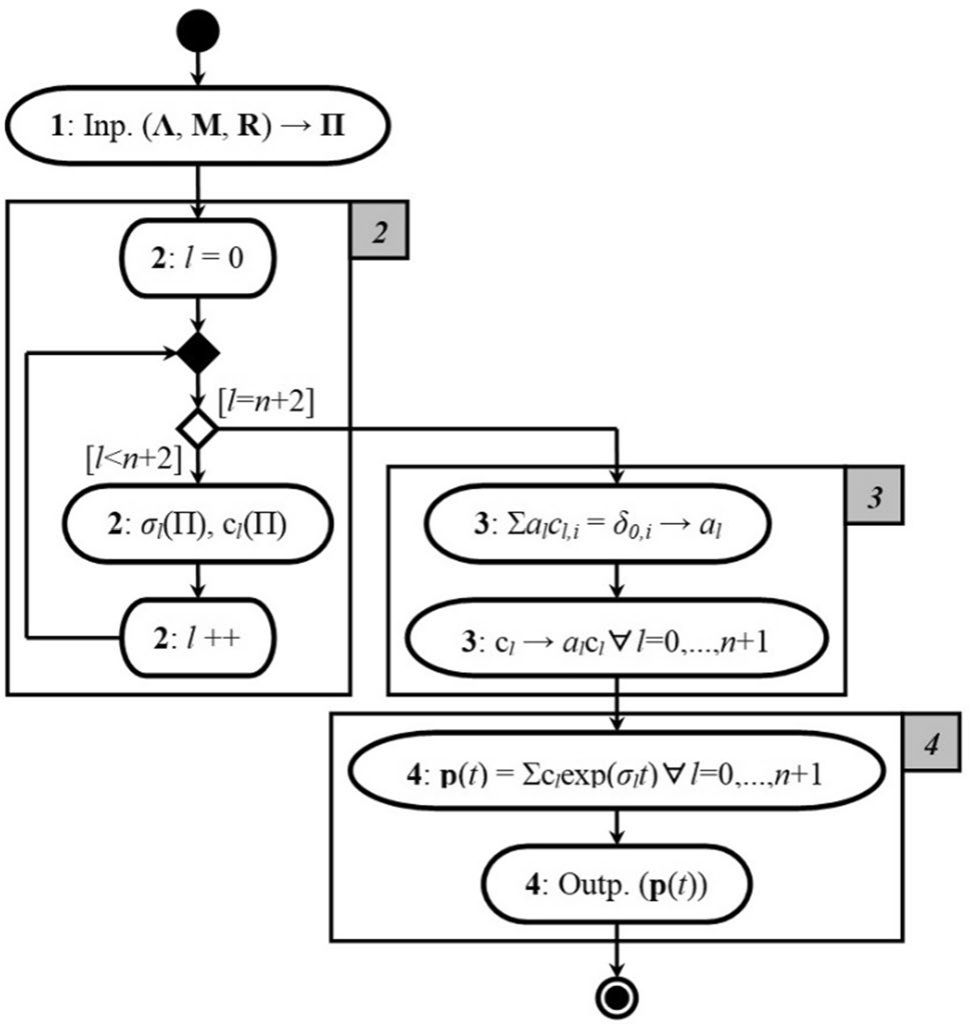
UML-activity diagram of the concept of solving the Chapman equation system for the model of the studied process.

The process of obtaining solutions of the Chapman eфiл of the form (1) with the initiating values (2) in the form of expression (9) can be easily generalized in the form of the UML-activity diagram presented in Fig. 2.

Let's comment on the stages presented in Fig. 2 computational process:

1. For the corresponding input parameters $\left\{ {\Lambda ,{\rm M},R} \right\}$ of the model (1), (2) we form a matrix of probabilities of transitions Π of the form (4);
2. We calculate the eigenvalues $\sigma_{l}$ and the eigenvectors ${\text{c}}_{l} = \left( {c_{l,i} } \right)$ of the matrix Π, $l = \overline{0,n + 1}$;
3. Solve the system of linear homogeneous equations $\sum\nolimits_{l = 0}^{n + 1} {a_{l} c_{l,i} } = \delta_{0,i}$ concerning the unknown $a_{l}$ and replace ${\text{c}}_{l} \to a_{l} {\text{c}}_{l} \forall l = \overline{0,n + 1}$;
4. According to expression (8) we obtain a formatted solution of the Cauchy problem for the system of Chapman's Eq. (1) with initiating values (2).

### Method of calculating indicators of reliability of the studied system taking into account the specifics of the process of its exploitation

Stochastic modeling is an effective tool that complements the process of rigorous analytical evaluation of the pool of quantitative characteristics (indicators) of reliability of the studied system, which, in the event of potential CPAs, is provided by the security subsystem.

Perhaps the most important and easy to interpret is such a reliability indicator as a time to failure (*TTF*). In the context of the mathematical model formulated in section “Research statement and the proposed model”, we define this indicator as the amount of time $T \in \left[ {0,\infty } \right)$ from the start of exploitation of the studied system ($t = 0$, the system is in the serviceable state *s*_0_) to the moment $t = T$ its transition to the state of failure *s*_*n*+1_ due to the successful CPA of any type.

We formally analyze the process of estimation of this indicator based on the Markov model of the security subsystem confrontation to the impact of typed CPAs. The indicator *T* is a continuous stochastic quantity. Define for a random stochastic quantity *T* the function and the density of the distribution as $F_{T} \left( t \right)$ and $f_{T} \left( t \right)$, respectively. The value of the function *F*_*T*_(*t*) at time *t* characterizes the probability that the value *T* will be less than or equal to *t*: $F_{T} \left( t \right) = P\left( {T \le t} \right)$, or (equivalently)—the probability that the studied system at a time *t* is in a state of failure $s_{n + 1}$: $F_{T} \left( T \right) = p_{n + 1} \left( t \right)$.

Since the function *F*_*T*_(*t*) is differentiated, equality $f_{T} \left( t \right) = F^{\prime}_{T} \left( t \right)$ holds. Therefore, we can write $f_{T} \left( t \right) = p^{\prime}_{n + 1} \left( t \right)$. Considering expression (9), the last expression for *f*_*T*_(*t*) can be rewritten as

11 $$f_{T} \left( T \right) = \sum\limits_{l = 1}^{n + 1} {c_{l,n} \sigma_{l} \exp \left( {\sigma_{l} t} \right)} .$$

If we take into account expression (11) in condition (10), we obtain the rationing condition:

$$\begin{aligned} \int\limits_{0}^{\infty } {f_{T} \left( t \right)dt} & = \sum\limits_{l = 1}^{n + 1} {c_{l,n + 1} \sigma_{l} \int\limits_{0}^{\infty } {\exp \left( {\sigma_{l} t} \right)dt} } \\ & = \sum\limits_{l = 1}^{n + 1} {c_{l,n + 1} \sigma_{l} \frac{1}{{\sigma_{l} }} = \sum\limits_{l = 1}^{n + 1} {c_{l,n + 1} } } = 1. \\ \end{aligned}$$

We present the process of calculating the *k*-first moments of the stochastic quantity *T* in terms of eigenvalues and eigenvectors of the matrix of transition probabilities (4). Interpret expressions (9) and (11) in the context of the definition of *k*-first moment:

12 $$\mu_{k} \left[ T \right] = \int\limits_{0}^{\infty } {t^{k} f_{T} \left( t \right)dt} = \sum\limits_{l = 1}^{n + 1} {c_{l,n + 1} \sigma_{l} \int\limits_{0}^{\infty } {t^{k} \exp \left( {\sigma_{l} t} \right)dt} } ,$$

where $c_{l,n + 1}$ is the $\left( {n + 1} \right)$-th element of the eigenvector ${\text{c}}_{l}$ of the matrix $\Pi$, equivalent to the eigenvalue $\sigma_{l}$.

Since $\sigma_{l} < 0\forall l = \overline{1,n + 1}$ the integrals in the right part of the expression (12) coincide:

13 $$\int\limits_{0}^{\infty } {t^{k} \exp \left( {\sigma_{l} t} \right)dt} = \frac{k!}{{\left| {\sigma_{l} } \right|^{k + 1} }}.$$

Substitute expression (13) into expression (12). The result is:

14 $$\mu_{k} \left[ T \right] = - \sum\limits_{l = 1}^{n + 1} {\frac{{c_{l,n + 1} }}{{\left| {\sigma_{l} } \right|^{k} }}} ,$$

In particular, based on expression (14), we define the mathematical expectation of the stochastic quantity *T* (or the time to the failure of the studied system) as

15 $$\tau = \mu_{1} \left[ T \right] = \sum\limits_{l = 1}^{n + 1} {\frac{{c_{l,n + 1} }}{{\sigma_{l} }}} .$$

Undoubtedly, the time to the failure of the studied system is a fundamental indicator of its reliability, provided by the security subsystem. Assessing the value of this indicator at the design stage of the latter will allow us to plan the number of funds for cyber-security measures adequately.

Using expression (15), we describe a fundamentally critical situation for the security subsystem when the latter lacks a protective mechanism to neutralize a certain CPA (equivalent situation—the security subsystem is disabled/absent).

In this situation, we have $r_{i} = 0\forall i \in \left\{ {\overline{1,n} } \right\}$. Accordingly, the eigenvalues of the matrix $\Pi$ will be equal to $\sigma_{0} = 0$, $\sigma_{1} = - \mu_{1}$, …, $\sigma_{n} = - \mu_{n}$, $\sigma_{n + 1} = - \lambda_{0}$. Determine the analytically corresponding eigenvectors ${\text{c}}_{l}$, $l = \overline{0,n + 1}$, that satisfy condition (7):

$$\begin{aligned} {\text{c}}_{0} & = \left( {0,0, \ldots ,0,1} \right), \\ {\text{c}}_{1} & = \left( {0,\frac{{\lambda_{1} }}{{\lambda_{0} - \mu_{1} }},0, \ldots ,0, - \frac{{\lambda_{1} }}{{\lambda_{0} - \mu_{1} }}} \right), \\ {\text{c}}_{2} & = \left( {0,0,\frac{{\lambda_{2} }}{{\lambda_{0} - \mu_{2} }},0, \ldots ,0, - \frac{{\lambda_{2} }}{{\lambda_{0} - \mu_{2} }}} \right), \\ & \quad \cdots \\ {\text{c}}_{n} & = \left( {0, \ldots ,0,\frac{{\lambda_{n} }}{{\lambda_{0} - \mu_{n} }}, - \frac{{\lambda_{n} }}{{\lambda_{0} - \mu_{n} }}} \right), \\ {\text{c}}_{n + 1} & = \left( {1, - \frac{{\lambda_{1} }}{{\lambda_{0} - \mu_{1} }}, - \frac{{\lambda_{2} }}{{\lambda_{0} - \mu_{2} }}, \ldots , - \frac{{\lambda_{n} }}{{\lambda_{0} - \mu_{n} }}, - 1 + \sum\limits_{j = 1}^{n} {\frac{{\lambda_{j} }}{{\lambda_{0} - \mu_{j} }}} } \right). \\ \end{aligned}$$

The defined set $\left\{ {\Sigma = \left( {\sigma_{0} ,\sigma_{1} , \ldots ,\sigma_{n + 1} } \right),{\text{C}} = \left( {{\text{c}}_{0} ,{\text{c}}_{1} , \ldots ,{\text{c}}_{n + 1} } \right)} \right\}$ allows us to formulate the desired expression for calculating the indicator $\tau$ based on expression (15):

16 $$\tau = \mu_{1} \left[ T \right] = \frac{1}{{\lambda_{0} }}\left( {1 + \sum\limits_{i = 1}^{n} {\frac{{\lambda_{i} }}{{\mu_{i} }}} } \right).$$

Assume that $n = 1$. Then, based on expression (16), we obtain $\tau = \lambda_{1}^{ - 1} + \mu_{1}^{ - 1}$, i.e., the average time to failure of the studied system $\tau$ is formed by the sum of the average time to CPA $\lambda_{1}^{ - 1}$ and the average time of its implementation $\mu_{1}^{ - 1}$.

**Figure 3:**
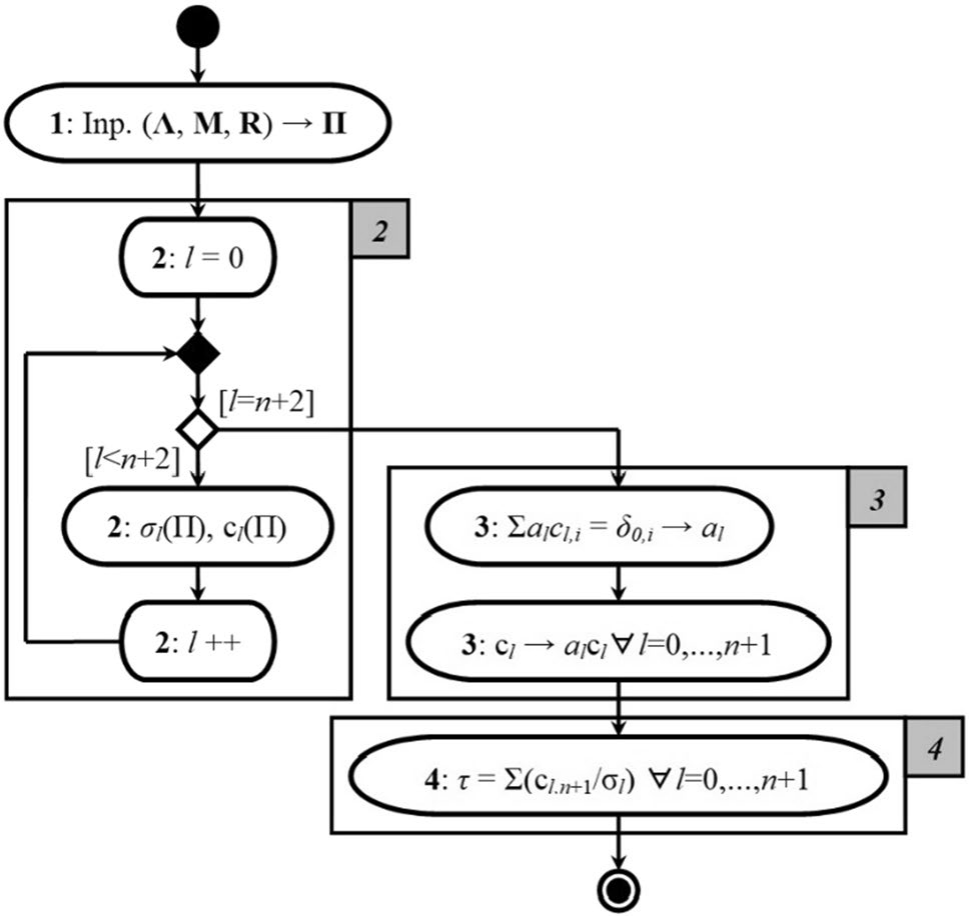
UML-activity diagram of the concept of calculating the time to failure of the studied system.

We summarize the proposed concept of calculating the time to failure as an indicator of the reliability of the studied system in the form of a UML-activity diagram, presented in Fig. 3.

Let's comment on the stages presented in Fig. 3 computational process:

1. For the corresponding input parameters $\left\{ {\Lambda ,{\rm M},R} \right\}$ of the model (1), (2) we form a matrix of probabilities of transitions Π of the form (4);
2. We calculate the eigenvalues $\sigma_{l}$ and the eigenvectors ${\text{c}}_{l} = \left( {c_{l,i} } \right)$ of the matrix Π, $l = \overline{0,n + 1}$;
3. Solve the system of linear homogeneous equations $\sum\nolimits_{l = 0}^{n + 1} {a_{l} c_{l,i} } = \delta_{0,i}$ concerning the unknown *a*_*l*_ and replace ${\text{c}}_{l} \to a_{l} {\text{c}}_{l} \forall l = \overline{0,n + 1}$;
4. According to expression (15), we calculate the time to failure of the studied system $\tau$.

## Experiments

As an example, we use the mathematical apparatus presented in section “Models and methods” to evaluate the reliability indicator of the real CPS of the Situation Center of the Information Technology Department of Vinnytsia City Council (Vinnytsia, Ukraine) (hereinafter—SC system). This info-communication system was put into operation in 2018 and is constantly evolving to improve the implemented services and add new ones. Currently, the SC system manages traffic lights on the roads of Vinnytsia. It maintains the uninterrupted exploitation of the data centre, which stores video streams from more than 1 k video cameras located in the city. Thus, the SC system is, by definition, cyber-physical.

The information collected in the SC system is confidential and available only to authorized employees of the Vinnytsia City Council, the National Police of Ukraine, the Security Service of Ukraine, etc. For these privileged persons to have quick access to the relevant information, a local network was created consisting of data centre servers, communication equipment, workstations, and software (i.e. SCADA). In normal exploitation, this LAN is not isolated from the Internet. However, the processing, storage, and audit of confidential information are carried out by a specialized relational database management system, access to which is organized through a specialized web interface. Data, databases, management systems, web interface—all these components are located on dedicated servers.

We describe the situation when attackers carry out a CPA on the SC system. Attackers seek information about network architecture, workstations, servers, operating systems, user accounts, etc. Analysis of this information can potentially identify hardware and software vulnerabilities, some of which may not fall within the scope of control of the protective mechanisms of the security subsystem. In the realities of modern cyberspace, exploits are often created based on data collected as a result of:$\lambda_{1}$ (Apache): analysis of internal and outgoing network traffic, remote access support mechanism; $\lambda_{2}$: buffer overflow; $\lambda_{3}$: SQL injection. Analysis of the logs of the SC system revealed the following categorized vulnerabilities: $\lambda_{1}$: (a) CVE-2019-9511, (b) CVE-2015-5206, (c) CVE-2019-9512, (d) CVE-2020-9481, (e) CVE-2020-17509; $\lambda_{2}$: (a) CVE-2008-0127, (b) CVE-2007-6593, (c) CVE-2021-36301, (d) CVE-2019-18805, (e) CVE-2017-6745; $\lambda_{3}$: (a) CVE-2021-45253, (b) CVE-2022-22055, (c) CVE-2021-45814, (d) CVE-2021-44599, (e) CVE-2020-0060. A full description of these vulnerabilities can be found at https://www.cvedetails.com/. Note that at the request of the Vinnytsia City Council administration, further in the set $\Lambda = \left\{ {\lambda_{1} ,\lambda_{2} ,\lambda_{3} } \right\}$ is not taken into account the entire list of vulnerabilities identified as a result of the analysis of logs of the SC system. However, these data are sufficient to demonstrate the functionality and prove the adequacy of the mathematical apparatus presented in section “Models and methods”.

Analysis of the logs of the SC system for the period from 01.09.2021 to 16.09.2021 (15 full days) in the context of detecting cases of CPAs described in the set $\Lambda$, allowed us to determine the following input data for modeling:

17 $$\begin{aligned} n & = 3,\quad \Lambda = \left( {4.27,3.96,1.12} \right), \\ {\rm M} & = \left( {0.91,0.41,0.94} \right),\quad R = \left( {0.09,0.39,0.0.37} \right). \\ \end{aligned}$$

Accordingly, the matrix $\Pi$ will look like this:

$$\Pi = \left( {\begin{array}{*{20}c} { - 9.381} & {4.27} & {3.96} & {1.12} & 0 \\ {0.093} & { - 0.91} & 0 & 0 & {0.827} \\ {0.169} & 0 & { - 0.41} & 0 & {0.251} \\ {0.362} & 0 & 0 & { - 0.94} & {0.588} \\ 0 & 0 & 0 & 0 & 0 \\ \end{array} } \right).$$

The spectrum (6) for this matrix will look like

$$spec\left( \Pi \right) = \left\{ {0, - 9.5461, - 0.9373, - 0.8528, - 0.3334} \right\}.$$

Applying the condition of normalization (7) of the form $\sum\nolimits_{l = 0}^{4} {c_{l} } = \left( {1,0,0,0,0} \right)$ to the eigenvectors ${\text{c}}_{{\text{l}}}$, $l = \overline{0,4}$, of the matrix $\Pi$ we obtain:

$$\begin{aligned} {\text{c}}_{0} & = \left( {0,0,0,0,1} \right); \\ {\text{c}}_{1} & = \left( {0.982, - 0.487, - 0.427, - 0.129,0.062} \right); \\ {\text{c}}_{2} & = \left( {0.001, - 0.064, - 0.002,0.023,0.042} \right); \\ {\text{c}}_{3} & = \left( {0.007,0.472, - 0.068,0.086, - 0.497} \right); \\ {\text{c}}_{4} & = \left( {0.011,0.079,0.497,0.020, - 0.606} \right). \\ \end{aligned}$$

According to expression (8) we obtain a formatted solution of the Cauchy problem for the above system of Chapman's equations and initiating values:

$$\begin{aligned} p_{0} \left( t \right) & = 0.982e^{ - 9.546t} + 0.001e^{ - 0.937t} + 0.007e^{ - 0.853t} + 0.011e^{ - 0.334t} \\ p_{1} \left( t \right) & = - 0.487e^{ - 9.456t} - 0.064e^{ - 0.937t} + 0.472e^{ - 0.853t} + 0.079e^{ - 0.334t} \\ p_{2} \left( t \right) & = - 0.427e^{ - 9.456t} - 0.002e^{ - 0.937t} - 0.068e^{ - 0.853t} + 0.497e^{ - 0.334t} \\ p_{3} \left( t \right) & = - 0.129e^{ - 9.456t} + 0.023e^{ - 0.937t} + 0.086e^{ - 0.853t} + 0.020e^{ - 0.334t} \\ p_{4} \left( t \right) & = 1 + 0.062e^{ - 9.456t} + 0.042e^{ - 0.937t} - 0.497e^{ - 0.853t} + 0.606e^{ - 0.334t} \\ \end{aligned}$$

**Figure 4:**
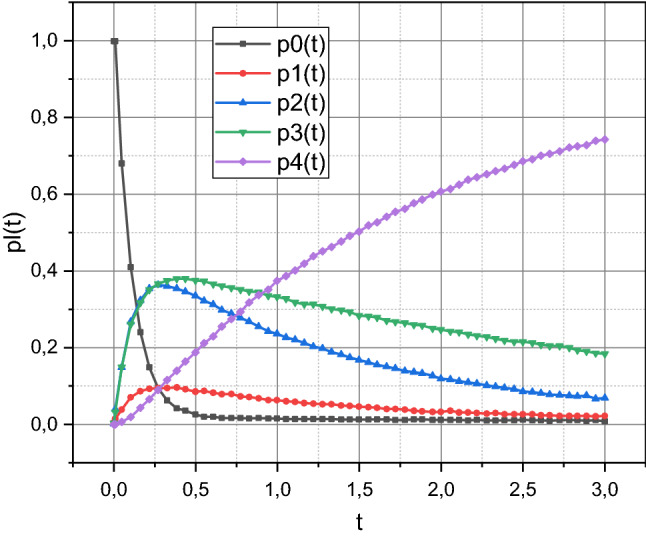
Dependences $p_{l} \left( t \right)$, $l = \overline{0,4}$, at $t \in \left[ {0,3} \right]$, calculated for the SC system using the concept presented in section “The concept of solving the Chapman equation system for the model of the studied process”.

Below, in Fig. 4, the dependences of the probabilities $p_{0} \left( t \right) \div p_{4} \left( t \right)$ at $t \in \left[ {0,3} \right]$ are visualized.

Now calculate the value of the time to failure s for the SC system. Since we have already given the results of the calculation of eigenvalues and eigenvectors of the matrix $\Pi$, which represents the model of the operation of the SC system, we can immediately apply the concept described in section “Method of calculating indicators of reliability of the studied system taking into account the specifics of the process of its exploitation”, summarized by expression (15):

$$\tau = \frac{0.062}{{ - 9.546}} + \frac{0.042}{{ - 0.937}} + \frac{ - 0.497}{{ - 0.853}} + \frac{ - 0.606}{{ - 0.334}} = 2.350.$$

Assuming that all elements of the set *R* are zero (protective mechanisms are completely helpless against the types of CPAs carried out on the SC system), then using expression (16) we can estimate the value of time to failure for the SC system:

$$\tau_{\min } \equiv \left. \tau \right|_{R = 0} = 1.737.$$

As expected, $\tau > \tau_{\min }$. The use of existing protective mechanisms (the effectiveness of which to counteract CPAs generalized by the set $\Lambda$ is represented by the values of the elements of the set *R*) allows extending the time to failure for the SC system by 35% relative to the estimated value of $\tau_{\min }$.

Thus, the concepts proposed in sections “The concept of solving the Chapman equation system for the model of the studied process” and “Method of calculating indicators of reliability of the studied system taking into account the specifics of the process of its exploitation”, based on the model presented in section “Research statement and the proposed model”, allow to visually, quantitatively, and computationally effectively characterize the reliability of a real CPS, considering the development of the process of the security subsystem countering to the impact of typed CPAs.

However, we still do not pay enough attention to the issue of proving the adequacy of the mathematical model of the security subsystem, countering the impact of typed CPAs presented in section “Research statement and the proposed model”. Let's fix this shortcoming.

We will compare the obtained empirical results for the SC system with simulation modeling results. To implement the simulation model, a specialized software MathWorks MATLAB was chosen. The rationale for this choice is that the toolbox functions of this software platform have been tested worldwide, and their adequacy has been empirically proven. Mainly using the Hidden Markov Model Toolbox, we created the following custom functions:

- $MyErlang\left( {k,\lambda } \right)$ is a function that simulates a stochastic quantity with Erlang distribution of the *k*-th order with a positive parameter $\lambda$;
- $MyState\left( {X,\Lambda ,{\rm M},R,k} \right)$ is a function for realizing the transition of the Markov chain in continuous time to the next state from the current *X*, which is characterized by a set $\left\langle {\Lambda ,{\rm M},R} \right\rangle$, where $\Lambda = \left\{ {\overline{{\lambda_{1} ,\lambda_{n} }} } \right\}$ is the set of CPAs flow intensities, $\lambda_{i} \ge 0$; ${\text{M}} = \left\{ {\overline{{\mu_{1} ,\mu_{n} }} } \right\}$ is the set of cyber-immune response flow intensities $\mu_{i} \ge 0$; $R = \left\{ {\overline{{r_{1} ,r_{n} }} } \right\}$ is the set of CPA neutralization probabilities, $0 \le r_{i} \le 1$;
- $MyTTF\left( {\Lambda ,{\rm M},R,k} \right)$ is a function for the implementation of a parameterized instance of a Markov chain in continuous time and the recognition for it of the time to failure through the consistent application of the concepts presented in sections “The concept of solving the Chapman equation system for the model of the studied process” and “Method of calculating indicators of reliability of the studied system taking into account the specifics of the process of its exploitation”.

Let us focus on the description of the function *MyState*. For a Markov chain, the state *X* is determined by a pair of parameters $\left( {t,m} \right)$, where *t* is the time of transition of the system to the state *X*; $m \in \left\{ {\overline{0,n + 1} } \right\}$ is the identifier of the state *X*. The function * MyState* implements the transition of the system from the current state *X *= (*t*, *m*) to the new state $X^{\prime } = \left( {t^{\prime } ,m^{\prime } } \right)$: $X \to X^{\prime }$. The values of the parameters $t^{\prime}$ $m^{\prime}$ are determined depending on the value of the parameter *m*. If:

- $m = 0$ (the system is in the serviceable state *s*_0_), the function *MyState* generates a set of stochastic quantities ${\rm T} = \left\{ {\tau_{i} } \right\}$, $i = \overline{1,n}$, distributed according to the Poisson distribution law with parameters $\lambda_{i}$, respectively. Let $\tau_{j} = \min \left\{ {\overline{{\tau_{1} ,\tau_{n} }} } \right\}$ be for $j \in \left\{ {\overline{1,n} } \right\}$, then take $t^{\prime} = t + \tau_{j}$ and $m^{\prime} = m + j$, i.e. $X^{\prime} = \left( {t + \tau_{j} ,j} \right)$;
- $m \in \left\{ {\overline{1,n} } \right\}$ (the system is in a state of counter-action to the *m*-th CPA $s_{m}$), the function *MyState* generates a stochastic quantity $\tau$, distributed according to the Poisson distribution law with the parameter $\mu_{m}$. We accept $t^{\prime} = t + \tau$. Using the standard MATLAB function $rand$, we obtain a stochastic number *x* evenly distributed over the interval $\left[ {0,1} \right]$. If the inequality $r_{m} > x$ holds, then we accept $m^{\prime} = 0$, otherwise we accept $m^{\prime} = n + 1$;
- $m = n + 1$ (the system is in a state of failure $s_{n + 1}$), then the function *MyState* takes $t^{\prime} = t$ and $m^{\prime} = m$. Accordingly, $X^{\prime} = X = \left( {t,m = n + 1} \right)$.

Operating according to the algorithm just described, the function *MyState* allows for a Markov chain in continuous time to implement a sequence of states of the form $X_{0} = \left( {t_{0} = 0,m_{0} = 0} \right)$ → $X_{1} = \left( {t_{1} ,m_{1} } \right)$ → … → $X_{M} = \left( {t_{M} ,M = n + 1} \right)$. Starting from the state *X*_0_, each implementation is generated by the function $MyTTF$. Transitions $X \to X^{\prime }$ occur until the implementation goes to the state $X_{M}$, $M \in {\mathbb{N}}$. It is *t*_*M*_ the value returned by the function *MyTTF*. It is the required time to failure of the system due to a successful CPA.

We compare the data calculated using the sequential application of the concepts presented in sections “The concept of solving the Chapman equation system for the model of the studied process” and “Method of calculating indicators of reliability of the studied system taking into account the specifics of the process of its exploitation” on time to failure of the SC system with the results of simulation modeling of the values of this reliability indicator obtained using the author's functions.

**Figure 5:**
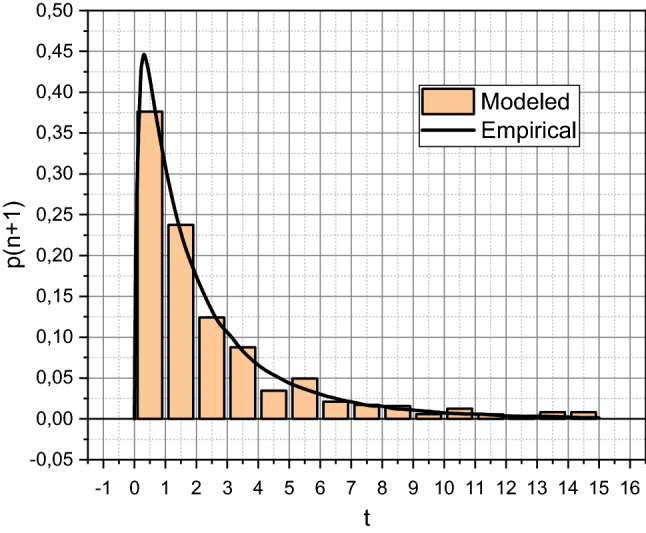
Proving the adequacy of the mathematical apparatus proposed in section “Models and methods”.

We obtained the last values by running the function $MyTTF$ with input parameters $\left\langle {\Lambda ,{\rm M},R} \right\rangle$ similar to (17). As a result, we obtained a set of 10^3^ simulated values of the time to failure for the SC system, which was visualized in the form of a histogram in Fig. 5. For clarity of comparison, we differentiated the empirically calculated function $p_{4} \left( t \right)$ for the SC system (see the analytical expression before Fig. 4), obtaining an empirical function of the probability density $f_{T} \left( T \right)$ of the form $f_{T} \left( t \right) = - 0.587e^{ - 9.546t} - 0.040e^{ - 0.937t} + 0.424e^{ - 0.853t} + 0.202e^{ - 0.334t}$ The obtained curve* f*_*T*_(*T*) is also visualized in Fig. 5.

**Figure 6:**
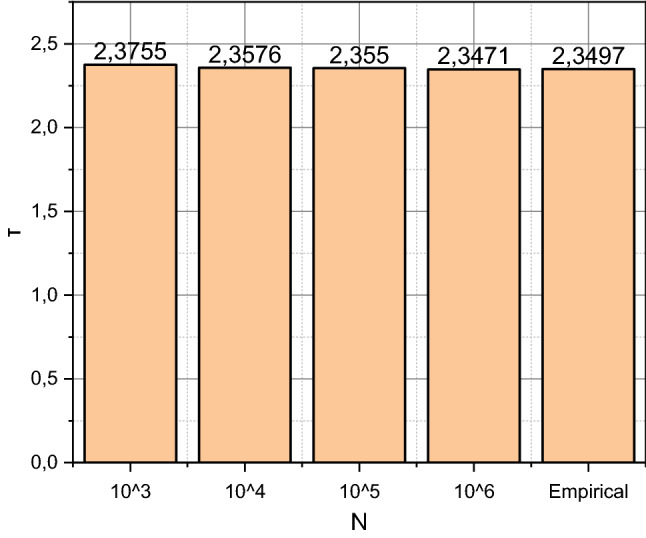
Precise comparison of empirical and simulated estimates of time to failure for the SC system.

From those presented in Fig. 5 results, it is obvious that the mathematical apparatus offered in section “Models and methods” is adequate. To further confirm this fact, we obtain estimates of the time to failure of the SC system by consistently generalizing the $N = \left( {10^{3} ,10^{4} ,10^{5} ,10^{6} } \right)$ results of statistical tests (starts of the author's function $MyTTF$). The obtained estimates are presented in the form of a diagram in Fig. 6.

Figure 6 shows that $10^{5} < N < 10^{6}$ empirical and simulated estimates of the SC system's time to failure (reliability indicator) coincide with the fourth decimal place.

## Discussion

The “cornerstone” of any research that results in a mathematical apparatus is to prove its adequacy. That is why we will start the discussion by analyzing the information presented in Figs. 5 and 6. In the first of them, we can compare the probability density functions of time to failure of the info-communication system of the Situational Center of the Department of Information Technology of Vinnytsia City Council (Vinnytsia, Ukraine), the results of exploitation of which were summarized as data in a set (17). Empirical values were calculated by sequential application of the concepts presented in sections “The concept of solving the Chapman equation system for the model of the studied process” and “Method of calculating indicators of reliability of the studied system taking into account the specifics of the process of its exploitation”, which allowed to obtain the shown in Fig. 5 smooth curve of the transition of the SC system to the state of failure. Showed in Fig. 5 histogram presents the simulation modeling process and summarizes the 10^3^ results of controlled experiments with an author's functions $MyTTF$, $MyState$ i $MyErlang$ implemented based on a priori adequate Hidden Markov Model Toolbox. It should be noted that the empirical and simulated results of estimation of the time to failure for the SC system coincide throughout the time interval of the analysis (for 15 full days). And this is even though the data visualized is characterized by significant nonlinearity, which persists throughout the censored interval of observations. Slight fluctuations of the simulated value relative to the empirical one in the general trend indicate a lack of accuracy in simulation modeling. This thesis is fully confirmed by the research results presented in Fig. 6. It is seen that 10^5^ < *N* < 10^6^ empirical and simulated estimates of the time to failure of the SC system coincide with the fourth decimal place. Thus, the adequacy of the mathematical apparatus proposed in the article is proved strictly following the provisions of the theory of experimental planning and mathematical statistics.

Now, starting from the research objectives, we need to demonstrate the functionality of the proposed mathematical apparatus because the question of assessing the reliability of automated systems has been studied for many decades. At once, we will note such advantages of the offered approach as simplicity and intelligibility. The analysis of the target info-communication system logs to detect the facts of exploitation of known vulnerabilities can be performed automatically. The mathematical apparatus proposed in the article allows evaluating the efficiency of the security subsystem in the form of a single indicator of reliability—time to failure, based on generalized sets of exploitation data in the form of a tuple $\left\langle {\Lambda ,{\rm M},R} \right\rangle$. Moreover, the proposed mathematical apparatus allows us to create a system-oriented reference point—to calculate the time to failure of the target system for a certain intensity of current CPAs when the security subsystem is disabled. A simple comparison of these two values indicates the effectiveness of the security subsystem. In particular, the increase in the time to failure for the SC system to activate the security subsystem in the context of current CPAs of type $\lambda_{1} \div \lambda_{3}$ was only 35%, prompting Vinnytsia City Council's guidance to allocate funds to purchase appropriate protective mechanisms.

Finally, we turn to the one shown in Fig. 4 empirical information. Note that $\sum\nolimits_{i = 0}^{4} {p_{i} \left( t \right)} = 1\forall t \in \left[ {0,3} \right]$ is an indirect confirmation of the adequacy of the created mathematical apparatus. As we noted in section “Models and methods”, the effectiveness of the security subsystem in counteracting typified CPAs increases with an increasing degree of convergence of the symmetric elements of the sets $\Lambda$ ${\rm M}$. Accordingly, in descending order of the probability of neutralization by the security subsystem, the types of CPAs should be arranged as follows: $\lambda_{3}$, $\lambda_{2}$, $\lambda_{1}$. In Fig. 4 we see that in descending order of absolute values, the curves $p_{i} \left( t \right)$, $i = \overline{1,3}$, are arranged in the following order: $p_{3} \left( t \right)$, $p_{2} \left( t \right)$, $p_{1} \left( t \right)$. Accordingly, experimental data confirmed the theoretical reasoning. Also, a significant discrepancy between the values of the sets Λ M causes a relatively small calculated value $\tau = 2.350$ for the security subsystem of the SC system.

## Conclusions

The article's main contribution is the description of the process of the security subsystem countering the impact of typed CPAs as a model of end states in continuous time. The input parameters of the model are the flow intensities of typed CPAs, the flow intensities of possible cyber-immune reactions, and the set of probabilities of neutralization of CPAs. The set of admissible states of the info-communication system is described taking into account possible variants of development of the modelled process. The initial parameters of the model are the probabilities of the studied system in the appropriate states at a certain moment. The dynamics of the life cycle of the info-communication system is embodied in the form of a matrix of transient probabilities. The mentioned matrix connects the initial parameters in the form of a system of Chapman's equations. The article presents a computationally efficient concept based on Gershgorin's theorems to solve such a system of equations with given initiating values. Based on the presented scientific results, the article proposes the concept of calculating the time to failure as an indicator of the reliability of the info-communication system operating under the probable impact of typical CPAs. The adequacy of the model and concepts presented in the article is proved by comparing a statically representative amount of empirical and simulated data. The application of the presented mathematical apparatus to analyze operational information of the Situational Center of the Information Technology Department of Vinnytsia City Council (Vinnytsia, Ukraine) showed the next. The security subsystem involved in the current CPAs prolongs the time to failure by 35% compared to the variant of operation of the mentioned info-communication system with an inactivated security subsystem.

Note that when formalizing the Markov model of the process of the security subsystem of the info-communication system confronting the impact of typed CPAs, it was considered that the latter ones are independent. The probable situation of simultaneous exploitation of one vulnerability by more than one attacker was also not considered. Considering these circumstances in the mathematical apparatus presented in the article is the direction of further research.

## Data Availability

Most data is contained within the article. All the data is available on request due to restrictions e.g., privacy or ethics.
